# Mechanisms Underlying Medication‐Related Osteonecrosis of the Jaw

**DOI:** 10.1111/odi.15198

**Published:** 2024-11-18

**Authors:** Kyeongho Lee, Kihun Kim, June Yeon Kim, Jin‐Woo Kim, Young‐Hoon Kang, Yun Hak Kim, Sung‐Jin Kim

**Affiliations:** ^1^ Department of Oral Histology and Developmental Biology, School of Dentistry and Dental Research Institute Seoul National University Seoul Korea; ^2^ Department of Biomedical Informatics, School of Medicine Pusan National University Yangsan Korea; ^3^ Department of Anatomy, School of Medicine Pusan National University Yangsan Korea; ^4^ Department of Oral and Maxillofacial Surgery, Research Institute for Intractable Osteonecrosis of the Jaw, College of Medicine Ewha Womans University Seoul Korea; ^5^ Department of Oral and Maxillofacial Surgery Changwon Gyeongsang National University Hospital, Gyeongsang National University School of Medicine Jinju Korea

## Abstract

**Objective:**

Medication‐related osteonecrosis of the jaw (MRONJ) is a rare but debilitating disease characterized by a progressive necrosis of jaw bones in patients who have received anti‐resorptive or anti‐angiogenic therapies. Unfortunately, we still have no validated preventive or pharmaceutical interventions to help these patients, primarily due to our limited understanding of MRONJ pathogenesis. Here, we offer an extensive review of recent studies relevant to MRONJ pathogenesis. We present a hypothesis regarding the coupling of bone resorption and angiogenesis that relies on osteoblast‐derived, matrix‐bound vascular endothelial growth factors to explain why ONJ is associated with both anti‐resorptive and anti‐angiogenic agents.

**Methods:**

A narrative review was conducted by searching databases, including PubMed, Scopus, Google Scholar, and Web of Science, to retrieve relevant reports.

**Results:**

Reduced bone resorption leads to reduced angiogenesis, and vice versa, creating a vicious cycle that ultimately results in ischemic necrosis of the jaw. Additionally, we suggest that reduced angiogenesis, induced by anti‐resorptive or anti‐angiogenic agents, aggravates bacterial infection‐induced bone necrosis, explaining why the jaw bone is particularly susceptible to necrosis.

**Conclusion:**

Our novel hypothesis will facilitate the advancement of future research and the development of more targeted approaches to managing MRONJ.

## 1 Introduction

Medication‐related osteonecrosis of the jaw (MRONJ) is a debilitating disease that often exposes the jaw bone in patients treated with anti‐resorptive therapy alone or in combination with anti‐angiogenic or immunomodulatory therapies (Ruggiero et al. 2022). These jaw‐related conditions significantly impact the quality of life (Kim et al. 2022). The current management guidelines for MRONJ include systemic antibiotic treatments and surgical removal of necrotic bone (Ruggiero et al. 2022), but there are no disease‐specific pharmaceutical interventions aimed at reducing surgical burden or improving prognosis. Moreover, the lack of preventive measures, which is largely due to our lack of understanding of MRONJ pathogenesis, can force patients requiring dental surgery to discontinue anti‐resorptive treatment for fear of reduced bone mineral density and increased fracture risk or even avoid dental surgery entirely (Ruggiero et al. 2022).

Although there are several hypotheses regarding MRONJ pathogenesis, the predominant view is that anti‐resorptive agents sometimes induce excessive suppression of bone remodeling (Allen and Burr 2009). This hypothesis does not, however, provide a mechanism by which reduced bone remodeling leads to osteonecrosis, nor does it explain ONJ associated with the combination of anti‐resorptive and anti‐angiogenic agents. Over the last decade, several groups have uncovered important molecular mechanisms underlying the healing of bone fractures and socket extractions. Just recently, a new opportunity to re‐evaluate MRONJ pathogenesis arose with the approval of the osteoporosis drug romosozumab, because it has both bone anabolic and anti‐resorptive effects.

Here, we present an extensive review of basic and clinical studies related to MRONJ pathogenesis. We present a novel hypothesis that explains the appearance of ONJ associated with anti‐resorptive and anti‐angiogenic agents via the coupling of bone resorption and angiogenesis. We suggest that reduced angiogenesis, induced by anti‐resorptive or anti‐angiogenic agents, aggravates bacterial infection‐induced bone necrosis, explaining why the jaw bone is particularly susceptible to necrosis. By elucidating these mechanisms, we aim to offer new insights that could guide future research and clinical approaches in the management of MRONJ.

## 2 Medications Associated With ONJ

### 2.1 Anti‐Resorptive Agents

Bisphosphonates (BPs) are drugs typically prescribed to treat osteoporosis and cancers with metastasis to the bone. BPs share two common phosphonate groups with high affinity for calcium ions, leading them to accumulate in calcium hydroxyapatite of bone tissues with a long skeletal half‐life (Khan et al. 1997). Under the acidic and enzyme‐rich microenvironment created by active osteoclasts, accumulated BPs are released from the bone matrix to enter nearby osteoclasts, inhibiting bone resorption and accelerating osteoclast apoptosis (Carano et al. 1990).

An association between BPs and osteonecrosis of the jaw (ONJ) was first reported in 2003 (Marx 2003). Because of its association with BP treatment, this condition was later dubbed bisphosphonate‐related ONJ (BRONJ) (Ruggiero, Fantasia, and Carlson 2006). The incidence of BRONJ in patients taking oral BPs for osteoporosis ranges from 0.01% to 0.06%, but the incidence of BRONJ in cancer patients receiving high‐dose intravenous BPs is 1%–8% (Anastasilakis et al. 2022).

Denosumab is a monoclonal antibody that targets the master regulator of osteoclast differentiation Receptor Activator of Nuclear Factor Kappa‐Β Ligand (RANKL). Denosumab was approved for use as an anti‐resorptive agent in 2010. By preventing the binding of RANKL to its receptor, denosumab disrupts the differentiation and survival of osteoclasts, suppressing osteoclastic bone resorption (Baron, Ferrari, and Russell 2011). Unexpectedly, denosumab has also been associated with the development of ONJ (Aghaloo, Felsenfeld, and Tetradis 2010). In addition, the incidence of denosumab‐related ONJ is generally higher than that of BRONJ in patients with osteoporosis (0.283% with denosumab vs. 0.045% with BPs) (Everts‐Graber et al. 2022) and cancer (approximately 1.5 times higher in meta‐analyses) (Chen et al. 2021).

### 2.2 Anti‐Angiogenic Agents

Anti‐angiogenic agents are mainly prescribed to cancer patients to restrict the tumor blood supply and suppress cancer cell migration through blood vessels (Carmeliet and Jain 2000). Angiogenesis is mediated by various signaling molecules, including vascular endothelial growth factor (VEGF), fibroblast growth factor 2 (FGF‐2), platelet‐derived growth factor (PDGF), and transforming growth factor‐beta (TGF‐β) (Klagsbrun and D'Amore 1991). Since VEGF is considered the master regulator of angiogenesis, VEGF and its downstream effectors are important targets for cancer treatment. Some VEGF inhibitors like bevacizumab and aflibercept bind directly to VEGF itself, while others like ramucirumab target VEGF receptor 2. More generic tyrosine kinase inhibitors, such as sunitinib, sorafenib, and pazopanib, can inhibit the phosphorylation and activation of receptor tyrosine kinases like the VEGF and PDGF receptors.

Given that ischemia, which is caused by a restriction of blood supply to living tissues, causes tissue necrosis, it is unsurprising that anti‐angiogenic drugs are also commonly associated with ONJ. MRONJ cases associated with anti‐angiogenic therapy in anti‐resorptive‐naïve patients are rare but consistently reported (Pimolbutr, Porter, and Fedele 2018). In one randomized controlled trial (RCT), bevacizumab‐related ONJ appeared in roughly 0.2% of BP‐naive cancer patients, compared to 0% in the placebo group (Guarneri et al. 2010). Other studies demonstrated an increased incidence of MRONJ in cancer patients treated simultaneously with BPs and anti‐angiogenic reagents (Beuselinck et al. 2012; Christodoulou et al. 2009).

Other classes of drugs associated with ONJ include the glucocorticoids, mTOR inhibitors, and various forms of chemotherapy (Anastasilakis et al. 2022). Although their pathogeneses in MRONJ are unclear, these drugs are commonly associated with anti‐angiogenic activity (Joussen et al. 1999; Karar and Maity 2011; Yano et al. 2006). Thus, in this paper, we focus on the pathogenesis of MRONJ associated with anti‐resorptive and anti‐angiogenic therapies. A list of anti‐resorptive and anti‐angiogenic agents associated with ONJ and their mechanisms of action are presented in Table 1.

**TABLE 1:** Representative medications associated with MRONJ and their mechanisms of action.

Class	Representative generic	Target	Mechanism of action
Anti‐resorptive agents			
Bisphosphonates	Alendronate, Risedronate, Ibandronate, Zoledronic acid	FPPS	Small molecule accumulated in extracellular bone matrix. Inhibits formation of metabolites essential for protein prenylation
RANKL antibody	Denosumab	RANKL	Monoclonal antibody of RANKL. Blocks the binding of RANKL to RANK receptor
Anti‐angiogenic agents			
VEGF inhibitors	Bevacizumab	VEGF‐A	Monoclonal antibody of VEGF‐A. Blocks the binding of VEGF‐A to VEGFR‐1 and VEGFR‐2
	Aflibercept	VEGFR ligands	Recombinant fusion protein consisting of VEGF‐binding portions from the extracellular domains of human VEGFR‐1 and ‐2. Blocks the binding of VEGFR ligands to VEGFR‐1 and ‐2
	Ramucirumab	VEGFR‐2	Monoclonal antibody of VEGFR2. Blocks the binding of VEGFR ligands to VEGFR‐2
TKIs	Sunitinib	VEGFRs, PDGFRs	Inhibits tyrosine phosphorylation and activation of target receptors
	Sorafenib	VEGFRs, PDGFRs	
	Pazopanib	VEGFRs, PDGFRs, FGFRs.	
	Cabozantinib	VEGFR‐2, c‐MET	
	Axitinib	VEGFRs, PDGFRs	

Abbreviations: FGFR, fibroblast growth factor receptor; FPPS, farnesyl diphosphate synthase; PDGFR, platelet‐derived growth factor receptor; RANKL, receptor activator of nuclear factor kappa‐Β ligand; VEGFR, vascular endothelial growth factor receptor.

## 3 MRONJ Pathogenesis: Reduced Bone Formation Versus Reduced Bone Resorption Caused by Anti‐Resorptive Agents

**FIGURE 1:**
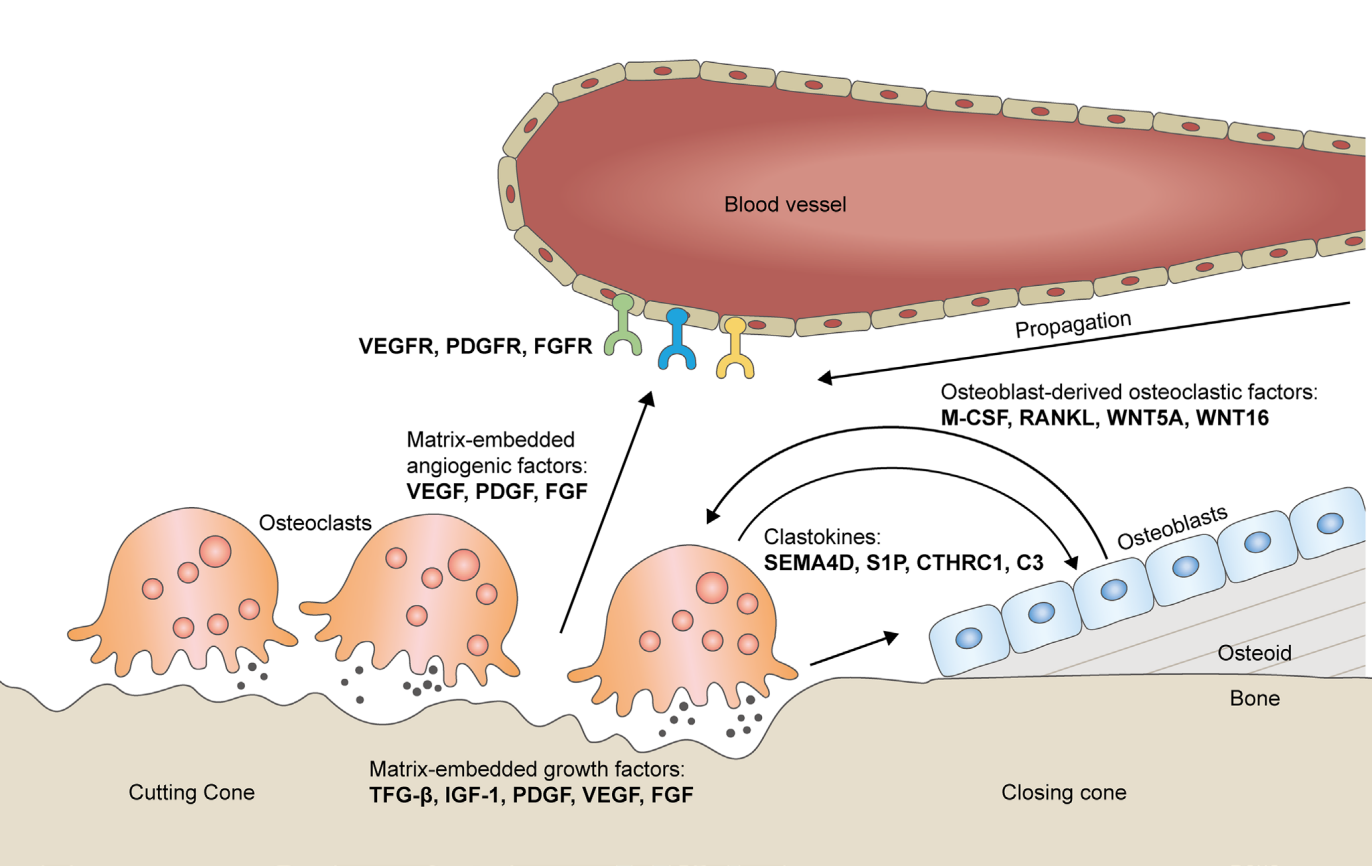
Schematic detailing osteoblast–osteoclast coupling and bone resorption–angiogenesis coupling in the basic multicellular unit of bone remodeling.

The most popular hypothesis regarding the pathogenesis of MRONJ points to excessive suppression of bone remodeling induced by anti‐resorptive agents (Allen and Burr 2009). Anti‐resorptive agents, such as BPs and denosumab, inhibit both osteoclastic bone resorption and osteoblastic bone formation due to osteoblast–osteoclast coupling. Anti‐resorptive agents reduce the release of osteogenic clastokines by inhibiting osteoclast differentiation; they reduce the release of osteogenic growth factors embedded in the bone matrix by decreasing bone resorption (Kim et al. 2020) (Figure 1). Therefore, suppression of bone remodeling reduces both bone resorption and formation. This raises the question of whether anti‐resorptive agents cause ONJ by suppressing bone resorption or formation.

The remodeling suppression hypothesis follows the idea that anti‐resorptive agents suppress bone remodeling to a greater extent in the alveolar process than the basal bone due to its higher rate of bone remodeling (Allen and Burr 2009). Both the rates of bone formation and resorption are higher in the alveolar process, probably to meet the functional demands of mastication. Treatment with anti‐resorptive agents should simultaneously affect both bone formation and resorption, so this phenomenon does not conclusively address the question.

Another line of evidence used to support the reduced bone formation hypothesis comes from the therapeutic effect of teriparatide in MRONJ patients. Teriparatide, which comprises the initial 34 amino acids of parathyroid hormone, promotes osteoblast‐mediated bone formation (Vall and Parmar 2023). Sim et al. demonstrated that patients treated with teriparatide showed increased MRONJ lesion resolution and improved serum levels of bone formation markers compared to patients treated with a placebo (Sim et al. 2020). However, teriparatide increases bone formation and resorption markers in both BP‐naive and BP‐pretreated patients, probably because of osteoblast–osteoclast coupling (Saag et al. 2007; Yoshiki et al. 2017). Moreover, even Sim et al. noted increased bone resorption markers in MRONJ patients treated with teriparatide (Sim et al. 2020). Thus, the therapeutic efficacy of teriparatide cannot resolve whether MRONJ arises because of reduced bone formation or resorption.

Romosozumab is a monoclonal antibody against sclerostin that has bone anabolic and anti‐resorptive effects and that was approved by FDA in 2019 for use in treating postmenopausal osteoporosis (McClung et al. 2014). If MRONJ is caused by reduced bone formation, romosozumab should theoretically reduce ONJ incidence. Although one rat study reported no incidence of ONJ after the administration of clinically relevant doses of romosozumab (Hadaya et al. 2019), rodents normally require much higher doses of anti‐resorptive agents to see MRONJ in studies with a limited number of animals (Yan et al. 2022). Unexpectedly, RCTs studying romosozumab reported three cases of MRONJ in the romosozumab groups and only one case in the control group (Table 2). Additionally, an analysis of FDA's Adverse Event Reporting System (FAERS) found that romosozumab treatment was associated with a slight but significant increase in the risk of MRONJ (Peng et al. 2022). These findings suggest that ONJ associated with anti‐resorptive agent treatment is likely caused by reduced bone resorption rather than reduced bone formation.

**TABLE 2:** Summary of the results of randomized controlled trials on romosozumab.

Author, year	Trial number	Study design	Age	Target population	No. of subjects	Intervention	Control	No. of ONJ cases
Cosman, 2016 (FRAME) (Cosman et al. 2016)	NCT01575834	Phase 3, multi‐center, randomized, double‐blind, parallel‐group, placebo‐controlled	55–90	Postmenopausal women with osteoporosis	Romosozumab (*n* = 3591) Placebo (*n* = 3589)	Romosozumab 210 mg subcutaneously once monthly for 12 months. After that, denosumab was given subcutaneously at a dose of 60 mg every 6 months for additional 12 months.	Placebo subcutaneously once monthly for 12 months. After that, denosumab was given subcutaneously at a dose of 60 mg every 6 months for additional 12 months.	2 in intervention group (*n* = 3581), 0 in control group (*n* = 3576)
Langdahl, 2017 (STRUCTURE) (Langdahl et al. 2017)	NCT01796301	Phase 3b, randomized, open‐label, active‐controlled, parallel‐group	55–90	Postmenopausal women with osteoporosis	Romosozumab (*n* = 218) Teriparatide (*n* = 218)	Romosozumab 210 mg subcutaneously once monthly for 12 months.	Teriparatide 20 μg subcutaneously once daily for 12 months.	No case of ONJ was reported
Saag, 2017 (ARCH) (Saag et al. 2017)	NCT01631214	Phase 3, multi‐center, international, randomized, double‐blind, alendronate‐controlled	55–90	Postmenopausal women with osteoporosis	Romosozumab (*n* = 2046) Alendronate (*n* = 2047)	Romosozumab 210 mg subcutaneously once monthly for 12 months. After that, alendronate is given orally at a dose of 70 mg every week for additional 24 months.	Alendronate 70 mg orally once a week for 12 months. After that, alendronate was given orally at a dose of 70 mg every week for additional 24 months.	1 in intervention group (*n* = 2040), 1 in control group (*n* = 2014)
Lewiecki, 2018 (BRIDGE) (Lewiecki et al. 2018)	NCT02186171	Phase 3, multi‐center, randomized, double‐blind, placebo‐controlled	55–90	Men with osteoporosis	Romosozumab (*n* = 163) Placebo (*n* = 82)	Romosozumab 210 mg subcutaneously once monthly for 12 months.	Placebo subcutaneously once monthly for 12 months.	No case of ONJ was reported
Baek, 2021 (Baek et al. 2021)	NCT02791516	Phase 3, multi‐center, randomized, double‐blind, placebo‐controlled	55–90	Postmenopausal women with osteoporosis	Romosozumab (*n* = 34) Placebo (*n* = 33)	Romosozumab 210 mg subcutaneously once monthly for 6 months.	Placebo subcutaneously once monthly for 6 months.	No case of ONJ was reported

## 4 Pathogenesis of MRONJ: Reduced Bone Resorption Versus Reduced Angiogenesis

**FIGURE 2:**
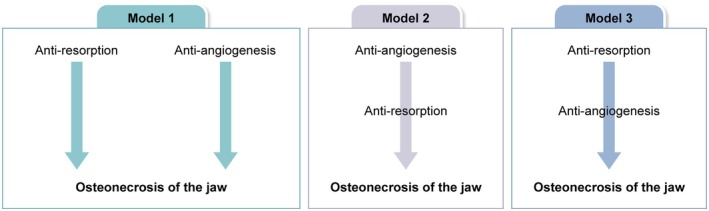
Schematic showing three scenarios regarding the link between anti‐resorptive and anti‐angiogenic agents and MRONJ.

Although MRONJ has a complex etiology, the link between anti‐resorptive and anti‐angiogenic agents and ONJ implicates reduced bone resorption and/or angiogenesis. This suggests three possible scenarios: Reduced bone resorption and angiogenesis are independently associated with MRONJ (Model 1); reduced angiogenesis leads to reduced bone resorption, resulting in MRONJ (Model 2); and reduced bone resorption leads to reduced angiogenesis, resulting in MRONJ (Model 3) (Figure 2).

Significant evidence links bone resorption and angiogenesis. First, treatment of cancer patients with zoledronic acid induces a significant reduction of circulating angiogenic factors, such as VEGF, PDGF, and TGF‐β (Ferretti et al. 2005; Santini et al. 2003). Animal model experiments demonstrated reduced neo‐vessel formation in the BRONJ group (Gao et al. 2021). Denosumab significantly inhibited angiogenesis in cancer patients (Girolami et al. 2016), and the denosumab mimic OPG‐Fc significantly reduced periodontal vascularity in mice (Gkouveris et al. 2019). Although Misso et al. suggested denosumab has no anti‐angiogenic activity (Misso et al. 2012), several factors suggest this is a misinterpretation of their results. First, they used a mouse model despite the fact that denosumab does not affect murine RANKL (Kostenuik et al. 2009). Second, the human umbilical vein endothelial cells (HUVECs) used in their in vitro experiments were inappropriate because of their low levels of RANKL transcript and because of conflicting reports regarding the role of RANKL in HUVECs (McGonigle, Giachelli, and Scatena 2009; Min et al. 2007). Moreover, any anti‐angiogenic activity of denosumab can only be properly assessed in vivo, as the major source of RANKL is bone tissue.

In contrast, angiogenic factors like VEGF, PDGF, and FGF‐2 directly stimulate osteoclast differentiation and function in vitro and in vivo (Chikazu et al. 2001; Li et al. 2017). Furthermore, VEGF inactivation by genetic deletion or pharmacologic inhibition suppresses osteoclastic bone resorption in vivo (Abu‐Amer et al. 2019; Kohno et al. 2005; Liu et al. 2012; Niida et al. 1999). Together, these data demonstrate a close association between bone resorption and angiogenesis, excluding Model 1.

Considering its low incidence and dose‐dependency, MRONJ seems to occur only when angiogenesis and/or bone resorption are severely disrupted. Anti‐angiogenic agents, such as sunitinib and sorafenib, reduced serum and urine levels of the bone resorption marker N‐terminal telopeptide (NTx) in cancer patients by roughly 40% (Dror Michaelson et al. 2009; Sahi et al. 2009), but this effect was much less pronounced than the effect of low‐dose BPs in patients with osteoporosis (Hadji et al. 2012). Given that MRONJ occurs in less than 0.1% of BP‐treated patients with osteoporosis, moderate reduction in bone resorption cannot explain the incidence of anti‐angiogenic agent‐related ONJ. Moreover, while the simultaneous use of anti‐resorptive and anti‐angiogenic agents increased the incidence of MRONJ (Beuselinck et al. 2012; Christodoulou et al. 2009), the combination of zoledronic acid and bevacizumab did not significantly alter serum levels of the bone resorption marker serum C‐terminal telopeptide compared to zoledronic acid alone (Francini et al. 2011). Together, these findings exclude Model 2.

Anti‐resorptive therapies with BPs or denosumab significantly reduced circulating angiogenic factors and vascularity in cancer patients (Girolami et al. 2016; Santini et al. 2003), but this was less effective than bevacizumab in terms of reducing circulating free VEGF (Loupakis et al. 2007). However, in humans, circulating VEGF levels are much lower than local tissue VEGF levels (Kut, Mac Gabhann, and Popel 2007). Moreover, local VEGF levels during wound healing typically rise several‐fold above circulating VEGF levels. This means the reduction in local VEGF levels observed in response to zoledronic acid was likely more dramatic than it seemed (Tamari et al. 2019). In addition, while bevacizumab barely affected the levels of angiogenic factors like PDGF and FGF‐2 (Madsen et al. 2012), zoledronic acid significantly reduced circulating levels of VEGF, PDGF, and FGF‐2 (Ferretti et al. 2005; Santini et al. 2003). Furthermore, anti‐resorptive and anti‐angiogenic agents synergistically improved cancer patient survival (Beuselinck et al. 2012), at least partly, because both have anti‐angiogenic effects. Therefore, these findings indicate that Model 3 is the most promising scenario. In it, anti‐resorption therapy causes ischemic ONJ by suppressing local angiogenesis. Because the basic multicellular unit responsible for bone remodeling comprises osteoclasts, osteoblasts, and microvessels, it is reasonable to expect a physiological coupling of bone resorption and angiogenesis (Figure 1).

## 5 Source of VEGF During the Healing of Extraction Sockets

Traumatic surgeries like tooth extraction are the strongest local risk factor for the development of MRONJ. After tooth extraction, acute inflammation occurs, followed by angiogenesis and the migration of mesenchymal stem cells (Vieira et al. 2015). Accordingly, local VEGF levels rise significantly after tooth extraction (Radovic et al. 2022), and disturbance of VEGF can delay the healing of extraction sockets (Abuohashish et al. 2019). These findings suggest that local elevation of VEGF in tooth extraction sockets is critical for normal healing. What, then, is the source of that local VEGF?

Several lines of evidence suggest osteoblast‐derived, bone‐matrix‐bound VEGF is the primary source of local VEGF during extraction socket healing. First, deletion of *Vegfa* in osteoblast lineage cells greatly reduced VEGF production at a site of bone repair in mice (Hu and Olsen 2016). VEGF was detected in osteoblasts rather than osteocytes in normal bone tissue and in a site of bone repair. Moreover, VEGF from early osteolineage cells, but not mature osteoblasts/osteocytes, plays a crucial role in angiogenesis and bone formation during fracture repair (Buettmann et al. 2019). This suggests osteoblasts and their precursor cells are an important source of VEGF in bone healing.

Second, the most prominent matrix‐bound isoforms of VEGF have strong pro‐angiogenic activity. Several isoforms of VEGFA arise from alternative mRNA splicing (Mamer, Wittenkeller, and Imoukhuede 2020), and the most abundant isoforms include VEGF189, VEGF165, and VEGF121. VEGF189 and VEGF165 are matrix‐bound because they have a heparin‐binding domain that allows interaction with the extracellular matrix, whereas VEGF121 is diffusible because it lacks the heparin‐binding domain. Matrix‐bound VEGF189 and VEGF165 have strong pro‐angiogenic activity, but VEGF121 has anti‐angiogenic or weak pro‐angiogenic activity (Mamer, Wittenkeller, and Imoukhuede 2020). Therefore, the pro‐angiogenic VEGFs produced in the extraction socket are likely matrix‐bound VEGFs.

Last, zoledronic acid reduces circulating VEGF levels even 21 days after treatment (Santini et al. 2003). Zoledronic acid accumulates primarily in the bone matrix, with its plasma concentration decreasing to 1% or less of its peak level by 24 h after infusion. This means the concentration of zoledronic acid in other tissues is negligible compared to bone (Shiraki et al. 2017; Weiss et al. 2008). Therefore, long‐lasting reductions in circulating VEGF are likely due to the reduced release of matrix‐bound VEGF from bone where zoledronic acid remains active. It is possible, however, that zoledronic acid also inhibits the production of VEGF from osteoblasts via osteoblast–osteoclast coupling. Together, these data indicate that the source of VEGF during the healing of extraction sockets is likely osteoblast‐derived, bone‐matrix‐bound VEGF.

## 6 Reduced Angiogenesis Aggravates Bacterial Infection‐Induced Bone Necrosis

The unique occurrence of MRONJ in the oral cavity is largely attributed to the fact that an extraction socket is an open wound, inviting infection by one or more of the approximately 700 species of oral bacteria (Deo and Deshmukh 2019). The risk of MRONJ increases when patients with periodontal disease undergo tooth extraction, suggesting oral bacterial infections contribute to MRONJ pathology (Kwoen et al. 2023). In the context of open fractures, bacterial infections impair bone healing, which exposes bone and surrounding tissues to the external environment (Johnson et al. 2007; Masters et al. 2022). During the initial inflammatory phase of wound healing, immune responses are activated to clear debris and recruit cells necessary for the healing process. Bacterial infections exacerbate this inflammatory response, leading to prolonged and excessive inflammation, which can damage healthy tissue and delay the healing. In addition, bacteria can form biofilms on exposed bone surfaces and protect them from antibiotics and the immune system. Such biofilms can also prevent osteoblasts from attaching to the bone surface, thereby hindering new bone production. Moreover, bacterial toxins can directly damage osteoblasts or alter their function, further impairing bone formation and leading to delayed or incomplete healing. Eventually, chronic infection can progress to osteomyelitis, a severe bone infection that destroys bone tissue.

Exposed bone in the extraction socket is particularly vulnerable to bacterial infection when patients take anti‐resorptive or anti‐angiogenic agents. This is because local vascularization is crucial for bacterial clearance after tooth extraction. Damaged tissues and bacterial contamination in the extraction socket trigger the release of pro‐inflammatory cytokines such as IL‐1, IL‐6, and TNF‐α (Udeabor et al. 2023). These mediators promote vasodilation and increase vascular permeability, allowing immune cells to migrate to the site of injury from circulation. Neutrophils are the first immune cells to arrive at the site, followed by macrophages and lymphocytes. They perform phagocytosis to engulf bacteria and necrotic tissue debris and release antimicrobial substances and enzymes to further combat infection. When local vascularization is reduced by anti‐resorptive or anti‐angiogenic agents, however, the initial recruitment of these immune cells and bacterial clearance are compromised. Additionally, reduced local vascularization leads to hypoxia, creating a favorable environment for the anaerobic bacteria that comprise the majority of species in MRONJ sites (Zirk et al. 2019). Hypoxia also impairs the function of recruited immune cells by reducing their survival, phagocytic activity, and production of reactive oxygen species (Krzywinska and Stockmann 2018). Together, the reduced angiogenesis secondary to treatment with anti‐resorptive or anti‐angiogenic agents aggravates bacterial infections in extraction sockets, leading to severe chronic inflammation.

## 7 A Model for MRONJ Pathogenesis: A Vicious Coupling of Bone Resorption and Angiogenesis Aggravates Bacterial Infection‐Induced Bone Necrosis

Growth factors produced by osteoblasts are readily embedded in unmineralized extracellular bone matrix and later released by osteoclastic bone resorption. This is the molecular basis of osteoblast–osteoclast coupling (Kim et al. 2020). Similarly, local bone resorption during the healing of extraction sockets promotes the release of angiogenic factors, such as VEGF, FGF‐2, and TGF‐β, embedded in the bone matrix (Kim et al. 2020; Lalani et al. 2005). This suggests that a coupling of bone resorption and angiogenesis could underly MRONJ. Such a model is supported by the finding that osteoclasts emerge at the socket walls and begin bone resorption 2 days after tooth extraction in rats (Smith 1974). It is also supported by the fourfold increase in salivary VEGF levels observed 3 days after tooth extraction in humans (Radovic et al. 2022).

Tooth extraction causes mechanical tissue damage and acute disruption of blood supply, which can lead to transient necrotic death of osteocytes and acute inflammation. In the normal healing of an extraction socket, this acute inflammation induces osteoclastic bone resorption. This removes necrotic bone tissue and releases trapped angiogenic factors, which then promote angiogenesis. However, when osteoclastic bone resorption is severely disrupted by anti‐resorptive agents, the angiogenic factors released from the bone matrix are insufficient to drive the angiogenesis required for normal healing. The resulting reduction in angiogenesis further inhibits local bone resorption, creating a vicious cycle that leads to the disruption of local vascularization.

**FIGURE 3:**
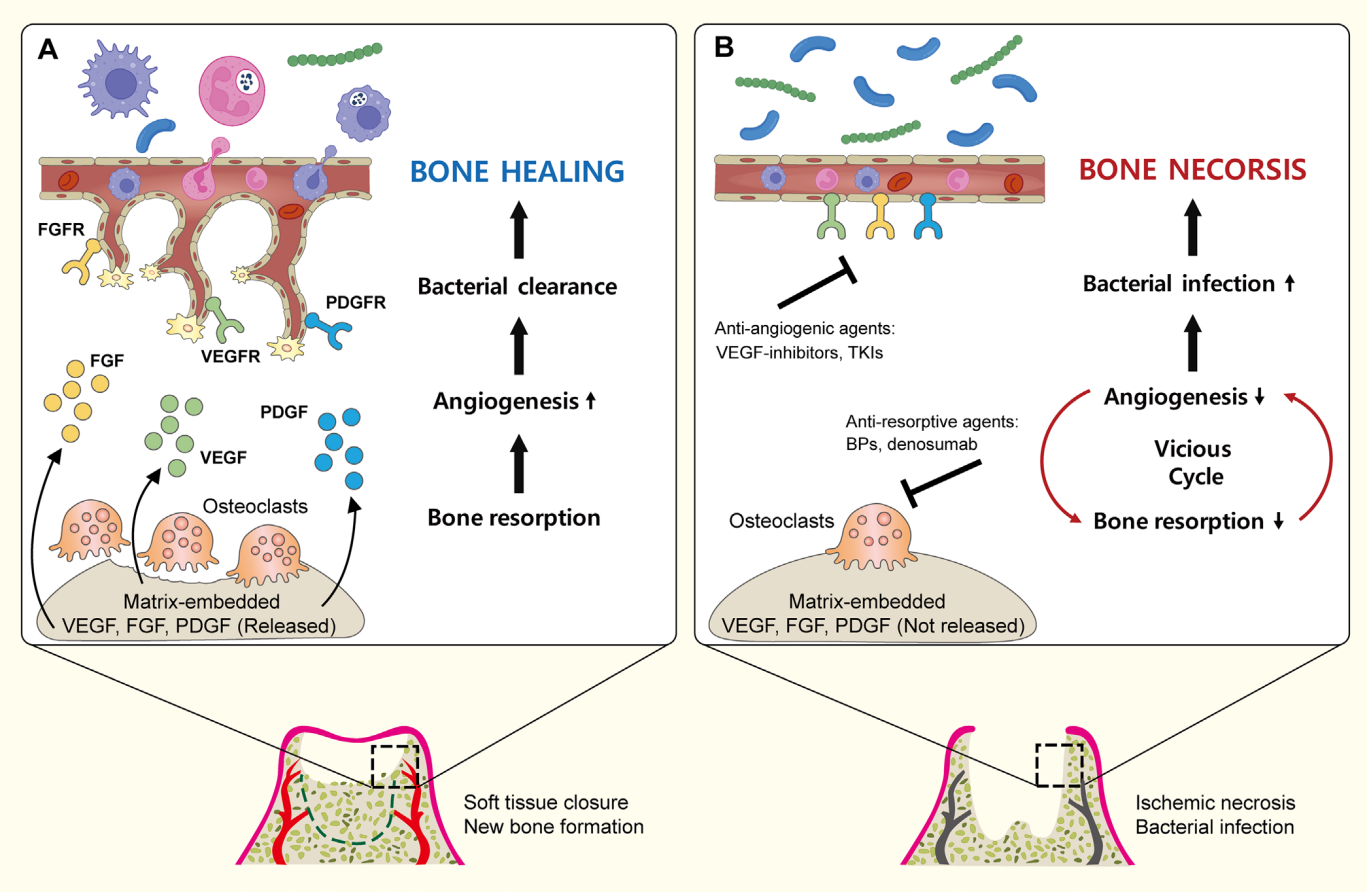
Schematic showing a model of MRONJ. (A) Normal healing of an extraction socket. (B) ONJ associated with anti‐resorptive or anti‐angiogenic agent treatment.

Because extraction sockets are open wounds and the oral cavity is a rich source of bacterial infection, bacterial contamination is inevitable during the healing of extraction sockets. The reduced local vascularization induced by anti‐resorptive or anti‐angiogenic agents aggravates bacterial infections by hindering the recruitment of immune cells and creating a hypoxic environment favorable for anaerobic oral bacteria and unfavorable for immune cell functions. This exacerbates bacterial infection, which, in turn, worsens bone necrosis. Thus, it is the coupling of bone resorption and angiogenesis that aggravates bacterial infection‐induced bone necrosis. A schematic detailing this mechanism appears in Figure 3.

## 8 Conclusions and Future Directions

The coupling of bone resorption and angiogenesis explains how ONJ can be associated with anti‐resorptive and/or anti‐angiogenic agents and suggests that anti‐resorptive agents cause ischemic necrosis of the jawbone via reduced bone resorption rather than reduced bone formation. We have highlighted the contribution of osteoblast‐derived, matrix‐bound VEGF in the healing of extraction sockets. Additionally, we suggest that reduced angiogenesis, induced by anti‐resorptive or anti‐angiogenic agents, aggravates bacterial infection‐induced bone necrosis, explaining why the jaw bone is particularly susceptible to necrosis. Based on this model, we propose that further investigation into the local delivery of bone‐resorptive agents and their role in enhancing local angiogenesis could provide new insights into preventing and managing MRONJ.

Although osteoblast‐derived, matrix‐bound VEGF is critical for the healing of extraction sockets, other angiogenic factors that contribute to local vascularization may not be restricted to the bone matrix. Future studies should identify any of these angiogenic factors that are associated with MRONJ as well as their sources. Larger prospective trials should clarify the causal relationship, epidemiology, and clinical characteristics of ONJ associated with romosozumab and anti‐angiogenic agent treatment. A recent study that took an integrated bioinformatic approach identified potential biomarkers as well as therapeutics for MRONJ, providing molecular insights for its diagnosis and treatment (Balachandran et al. 2023). The therapeutic interventions they suggested, as well as those we propose here, should be verified in preclinical and clinical studies to determine their efficacy and complications.

## Author Contributions

**Kyeongho Lee:** data curation, formal analysis, writing – original draft. **Kihun Kim:** data curation, formal analysis, writing – original draft. **June Yeon Kim:** investigation. **Jin‐Woo Kim:** investigation, writing – review and editing. **Young‐Hoon Kang:** investigation, writing – review and editing. **Yun Hak Kim:** conceptualization, investigation, writing – review and editing, funding acquisition. **Sung‐Jin Kim:** conceptualization, investigation, writing – review and editing, funding acquisition.

## Conflicts of Interest

The authors declare no conflicts of interest.

## Data Availability

Data sharing is not applicable to this article as no new data were created or analyzed in this study.
